# Current Awareness on Comparative and Functional Genomics

**DOI:** 10.1002/cfg.355

**Published:** 2004


**Comparative and Functional Genomics**
*Comp Funct Genom* 2004; **5**: 555–562
Published online in Wiley InterScience (www.interscience.wiley.com). **DOI:** 10.1002cfg.355

### Current awareness on comparative and functional genomics

CURRENT AWARENESS - USE THE LINKS ONLINE

### 1 Reviews & symposia

Apweiler R, Bairoch A, Wu CH. 2004. Protein sequence databases. *Curr Opin Chem Biol* **8:** (1) 76.

Bestel-Corre G, Dumas-Gaudot E, Gianinazzi S. 2004. Proteomics as a tool to monitor plant-microbe endosymbioses in the rhizosphere. *Mycorrhiza* **14:** (1) 1.

Buchanan-Wollaston V, Earl S, Harrison E, Mathas E, Navabpour S, Page T, Pink D. 2003. The molecular analysis of leaf senescence - A genomics approach. *Plant Biotechnol J* **1:** (1) 3.

Canovas FM, Dumas-Gaudot E, Recorbet G, Jorrin J, Mock HP, Rossignol M. 2004. Plant proteome analysis. *Proteomics* **4:** (2) 285.

Davis TN. 2004. Protein localization in proteomics. *Curr Opin Chem Biol* **8:** (1) 49.

Delrue RM, Lestrate P, Tibor A, Letesson JJ, De Bolle X. 2004. *Brucella* pathogenesis, genes identified from random large-scale screens. *FEMS Microbiol Lett* **231:** (1) 1.

Feldman AL, Espina V, Petricoin EF, Liotta LA, Rosenblatt KP. 2004. Use of proteomic patterns to screen for gastrointestinal malignancies. *Surgery* **135:** (3) 243.

Firsov D. 2004. Revisiting sodium and water reabsorption with functional genomics tools. *Curr Opin Nephrol Hypertens* **13:** (1) 59.

Gerner C. 2004. Screening for disease-markers and investigating drug effects by proteome profiling: Can it meet expectations? *Comb Chem High Throughput Scr* **7:** (1) 1.

Gerstein M, Echols N. 2004. Exploring the range of protein flexibility, from a structural proteomics perspective. *Curr Opin Chem Biol* **8:** (1) 14.

Goodman AL, Lory S. 2004. Analysis of regulatory networks in *Pseudomonas aeruginosa* by genome wide transcriptional profiling. *Curr Opin Microbiol* **7:** (1) 39.

Halapi E, Hakonarson H. 2004. Recent development in genomic and proteomic research for asthma. *Curr Opin Pulm Med* **10:** (1) 22.

Herrgard MJ, Covert MW, Palsson BO. 2004. Reconstruction of microbial transcriptional regulatory networks. *Curr Opin Biotechnol* **15:** (1) 70.

Holzman T, Kolker E. 2004. Statistical analysis of global gene expression data: Some practical considerations. *Curr Opin Biotechnol* **15:** (1) 52.

Hubank M. 2004. Gene expression profiling and its application in studies of haematological malignancy. *Br J Haematol* **124:** (5) 577.

Jensen ON. 2004. Modification-specific proteomics: Characterization of post-translational modifications by mass spectrometry. *Curr Opin Chem Biol* **8:** (1) 33.

Jessani N, Cravatt BF. 2004. The development and application of methods for activity-based protein profiling. *Curr Opin Chem Biol* **8:** (1) 54.

Jones S, Thornton JM. 2004. Searching for functional sites in protein structures. *Curr Opin Chem Biol* **8:** (1) 3.

Kasinathan C, Vrana K, Beretta L, Thomas P, Gooch R, Worst T, Walker S, Xu A, Pierre P, Green H *et al*. 2004. The future of proteomics in the study of alcoholism. *Alcohol Clin Exp Res* **28:** (2) 228.

Kazazian HH. 2004. Mobile elements: Drivers of genome evolution. *Science* **303:** (5664) 1626.

Knox DP. 2004. Technological advances and genomics in metazoan parasites. *Int J Parasitol* **34:** (2) 139.

Kortemme T, Baker D. 2004. Computational design of protein-protein interactions. *Curr Opin Chem Biol* **8:** (1) 91.

Liepman AH, Olsen LI. 2004. Genomic analysis of aminotransferases in *Arabidopsis thaliana*. *Crit Rev Plant Sci* **23:** (1) 73.

Mantripragada KK, Buckley PG, De Stahl TD, Dumanski JP. 2004. Genomic microarrays in the spotlight. *Trends Genet* **20:** (2) 87.

McCammon MG, Robinson CV. 2004. Structural change in response to ligand binding. *Curr Opin Chem Biol* **8:** (1) 60.

McManus DP, Le TH, Blair D. 2004. Genomics of parasitic flatworms. *Int J Parasitol* **34:** (2) 153.

Meloni R, Khalfallah O, Biguet NF. 2004. DNA microarrays and pharmacogenomics. *Pharmacol Res* **49:** (4) 303.

Michnick SW. 2004. Proteomics in living cells. *Drug Discov Today* **9:** (6) 262.

Mitsopoulos G, Walsh DP, Chang YT. 2004. Tagged library approach to chemical genomics and proteomics. *Curr Opin Chem Biol* **8:** (1) 26.

Navas A, Albar JP. 2004. Application of proteomics in phylogenetic and evolutionary studies. *Proteomics* **4:** (2) 299.

Nesvizhskii AI, Aebersold R. 2004. Analysis, statistical validation and dissemination of large-scale proteomics datasets generated by tandem MS. *Drug Discov Today* **9:** (4) 173.

Papin J, Subramaniam S. 2004. Bioinformatics and cellular signaling. *Curr Opin Biotechnol* **15:** (1) 78.

Patil KR, Akesson M, Nielsen J. 2004. Use of genome-scale microbial models for metabolic engineering. *Curr Opin Biotechnol* **15:** (1) 64.

Pearlberg J, LaBaer J. 2004. Protein expression clone repositories for functional proteomics. *Curr Opin Chem Biol* **8:** (1) 98.

Petricoin EF, Liotta LA. 2004. Proteomic approaches in cancer risk and

In order to keep subscribers up-to-date with the latest developments in their field, this current awareness service is provided by John Wiley & Sons and contains newly-published material on comparative and functional genomics. Each bibliography is divided into 16 sections. 1 Reviews & symposia; 2 General; 3 Large-scale sequencing and mapping; 4 Evolutionary genomics; 5 Comparative genomics; 6 Pathways, gene families and regulons; 7 Pharmacogenomics; 8 EST, cDNA and other clone resources; 9 Functional genomics; 10 Transcriptomics; 11 Proteomics; 12 Protein structural genomics; 13 Metabolomics; 14 Genomic approaches to development; 15 Technological advances; 16 Bioinformatics. Within each section, articles are listed in alphabetical order with respect to author. If, in the preceding period, no publications are located relevant to any one of these headings, that section will be omitted.

Copyright © 2004 John Wiley & Sons, Ltd.

response assessment. *Trends Mol Med* **10:** (2) 59.
Petricoin EF, Liotta LA. 2004. SELDI-TOF based serum proteomic pattern diagnostics for early detection of cancer. *Curr Opin Biotechnol* **15:** (1) 24.
Pignatti PF. 2004. Trends in pharmacogenomics of drugs used in the treatment of asthma. *Pharmacol Res* **49:** (4) 343.
Pirazzoli A, Recchia G. 2004. Pharmacogenetics and pharmacogenomics: are they still promising? *Pharmacol Res* **49:** (4) 357.
Predki PF. 2004. Functional protein microarrays: Ripe for discovery. *Curr Opin Chem Biol* **8:** (1) 8.
Rual JF, Hill DE, Vidal M. 2004. ORFeome projects: Gateway between genomics and omics. *Curr Opin Chem Biol* **8:** (1) 20.
Sauer U. 2004. High-throughput phenomics: Experimental methods for mapping fluaxomes. *Curr Opin Biotechnol* **15:** (1) 58.
Schnable PS, Hochholdinger F, Nakazono M. 2004. Global expression profiling applied to plant development. *Curr Opin Plant Biol* **7:** (1) 50.
Sharom JR, Bellows DS, Tyers M. 2004. From large networks to small molecules. *Curr Opin Chem Biol* **8:** (1) 81.
Sheffield LG, Gavinski JJ. 2003. Proteomics methods for probing molecular mechanisms in signal transduction. *J Anim Sci* **81:** (Suppl 3) 48.
Speers AE, Cravatt BF. 2004. Chemical strategies for activity-based proteomics. *Chembiochem* **5:** (1) 41.
Steinmetz LM, Davis RW. 2004. Maximizing the potential of functional genomics. *Nat Rev Genet* **5:** (3) 190.
Toure YT, Oduola AMJ, Morel CM. 2004. The *Anopheles gambiae* genome: Next steps for malaria vector control. *Trends Parasitol* **20:** (3) 142.
Trotta R, Donati MB, Iacoviello L. 2004. Trends in pharmacogenomics of drugs acting on hypertension. *Pharmacol Res* **49:** (4) 351.
Van den Bergh G, Arckens L. 2004. Fluorescent two-dimensional difference gel electrophoresis unveils the potential of gel-based proteomics. *Curr Opin Biotechnol* **15:** (1) 38.
Veerasingham SJ, Sellers KW, Raizada MK. 2004. Functional genomics as an emerging strategy for the investigation of central mechanisms in experimental hypertension. *Prog Biophys Mol Biol* **84:** (2-3) 107.
Wackett LP, Dodge AG, Ellis LBM. 2004. Microbial genomics and the periodic table. *Appl Environ Microbiol* **70:** (2) 647.
Walduck A, Rudel T, Meyer TF. 2004. Proteomic and gene profiling approaches to study host responses to bacterial infection. *Curr Opin Microbiol* **7:** (1) 33.
Wilson KE, Ryan MM, Prime JE, Pashby DP, Orange PR, O'Beirne G, Whateley JG, Bahn S, Morris CM. 2004. Functional genomics and proteomics: Application in neurosciences. *J Neurol Neurosurg Psychiatry* **75:** (4) 529.
Yakunin AF, Yee AA, Savchenko A, Edwards AM, Arrowsmith CH. 2004. Structural proteomics: A tool for genome annotation. *Curr Opin Chem Biol* **8:** (1) 42.
Zhang H, Yan W, Aebersold R. 2004. Chemical probes and tandem mass spectrometry: A strategy for quantitative analysis of proteomes and subproteomes. *Curr Opin Chem Biol* **8:** (1) 66.

### 3 Large-scale sequencing and mapping

Antonopoulos DA, Nelson KE, Morrison M, White BA. 2004. Strain-specific genomic regions of *Ruminococcus flavefaciens* FD-1 as revealed by combinatorial random-phase genome sequencing and suppressive subtractive hybridization. *Environ Microbiol* **6:** (4) 335.
Castelli V, Aury JM, Jaillon O, Wincker P, Clepet C, Menard M, Cruaud C, Quetier F, Scarpelli C, Schachter V *et al*. 2004. Whole genome sequence comparisons with "full-length" cDNA sequences: A combined approach to evaluate and improve *Arabidopsis* genome annotation. *Genome Res* **14:** (3) 406.
Garcia-Chapa M, Batlle A, Rekab D, Rosquete MR, Firrao G. 2004. PCR-mediated whole genome amplification of phytoplasmas. *J Microbiol Methods* **56:** (2) 231.
Istrail S, Sutton GG, Florea L, Halpern AL, Mobarry CM, Lippert R, Walenz B, Shatkay H, Dew I, Miller JR *et al*. 2004. Whole-genome shotgun assembly and comparison of human genome assemblies. *Proc Natl Acad Sci U S A* **101:** (7) 1916.
Kato T, Sato S, Nakamura Y, Kaneko T, Asamizu E, Tabata S. 2003. Structural analysis of a *Lotus japonicus* genome. V. Sequence features and mapping of sixty-four TAC clones which cover the 6.4 Mb regions of the genome. *DNA Res* **10:** (6) 277.
Pridmore RD, Berger B, Desiere F, Vilanova D, Barretto C, Pittet AC, Zwahlen MC, Rouvet M, Altermann E, Barrangou R *et al*. 2004. The genome sequence of the probiotic intestinal bacterium *Lactobacillus johnsonii* NCC 533. *Proc Natl Acad Sci U S A* **101:** (8) 2512.
Thangavelu M, James AB, Bankier A, Bryan GJ, Dear PH, Waugh R. 2003. HAPPY mapping in a plant genome: Reconstruction and analysis of a high-resolution physical map of a 1.9 Mbp region of *Arabidopsis thaliana* chromosome 4. *Plant Biotechnol J* **1:** (1) 23.
Westberg J, Persson A, Holmberg A, Goesmann A, Lundeberg J, Johansson KE, Pettersson B, Uhlen M. 2004. The genome sequence of *Mycoplasma mycoides* subsp *mycoides* SC type strain $PG1^{T}$, the causative agent of contagious bovine pleuropneumonia (CBPP). *Genome Res* **14:** (2) 221.
Wu CC, Sun SK, Nimmakayala P, Santos FA, Meksem K, Springman R, Ding K, Lightfoot DA, Zhang HB. 2004. A BAC and BIBAC-based physical map of the soybean genome. *Genome Res* **14:** (2) 319.

### 4 Evolutionary genomics

Chu KH, Qi J, Yu ZG, Anh V. 2004. Origin and phylogeny of chloroplasts revealed by a simple correlation analysis of complete genomes. *Mol Biol Evol* **21:** (1) 200.
Neff BD. 2004. Stabilizing selection on genomic divergence in a wild fish population. *Proc Natl Acad Sci U S A* **101:** (8) 2381.
Vandepoele K, De Vos W, Taylor JS, Meyer A, Van de Peer Y. 2004. Major events in the genome evolution of vertebrates: Paranome age and size differ considerably between ray-finned fishes and land vertebrates. *Proc Natl Acad Sci U S A* **101:** (6) 1638.
Wong CW, Albert TJ, Vega VB, Norton JE, Cutler DJ, Richmond TA, Stanton LW, Liu ET, Miller LD. 2004. Tracking the evolution of the SARS coronavirus using high-throughput, high-density resequencing arrays. *Genome Res* **14:** (3) 398.
Yeh SH, Wang HY, Tsai CY, Kao CL, Yang JY, Liu HW, Su IJ, Tsai SF, Chen DS, Chen PJ. 2004. Characterization of severe acute respiratory syndrome coronavirus genomes in Taiwan: Molecular epidemiology and genome evolution. *Proc Natl Acad Sci U S A* **101:** (8) 2542.

### 5 Comparative genomics

Blatny JM, Godager L, Lunde M, Nes IF. 2004. Complete genome sequence of the *Lactococcus lactis* temperature phage ϕLC3: Comparative analysis of φiLC3 and its relatives in lactococci and streptococci. *Virology* **318:** (1) 231.
Cushion MT. 2004. Comparative genomics of *Pneumocystis carinii* with other protists: Implications for life style. *J Eukaryot Microbiol* **51:** (1) 30.
Liu YY, Liu XS, Wei LP, Altman RB, Batzoglou S. 2004. Eukaryotic regulatory element conservation analysis and identification using comparative genomics. *Genome Res* **14:** (3) 451.
Mitreva M, McCarter JP, Martin J, Dante M, Wylie T, Chiapelli B, Pape D, Clifton SW, Nutman TB, Waterston RH. 2004. Comparative genomics of gene expression in the parasitic and free-living nematodes *Strongyloides stercoralis* and *Caenorhabditis elegans*. *Genome Res* **14:** (2) 209.
Nowrousian M, Wurtz C, Poggeler S, Kuck U. 2004. Comparative sequence analysis of *Sordaria macrospora* and *Neurospora crassa* as a means to improve genome annotation. *Fungal Genet Biol* **41:** (3) 285.
Shibuya K, Kudoh J, Obayashi I, Shimizu A, Sasaki T, Minoshima S, Shimizu N. 2004. Comparative genomics of the keratin-associated protein (KAP) gene clusters in human, chimpanzee, and baboon. *Mamm Genome* **15:** (3) 179.
Uddin M, Wildman DE, Liu GZ, Xu WB, Johnson RM, Hof PR, Kapatos G, Grossman LI, Goodman M. 2004. Sister grouping of chimpanzees and humans as revealed by genome-wide phylogenetic analysis of brain gene expression profiles. *Proc Natl Acad Sci U S A* **101:** (9) 2957.

### 6 Pathways, gene families and regulons

Berns K, Hijmans EM, Mullenders J, Brummelkamp TR, Velds A, Heimerikx M, Kerkhoven RM, Meadiredjo M, Nijkamp W, Weigelt B *et al*. 2004. A large-scale RNAi screen in human cells identifies new

Copyright © 2004 John Wiley & Sons, Ltd. 

components of the p53 pathway. *Nature* **428:** (6981) 431.
Leverrier P, Vissers JPC, Rouault A, Boyaval P, Jan G. 2004. Mass spectrometry proteomic analysis of stress adaptation reveals both common and distinct response pathways in *Propionibacterium freudenreichii*. *Arch Microbiol* **181:** (3) 215.
Marmiesse M, Brodin P, Buchrieser C, Gutierrez C, Simoes N, Vincent V, Glaser P, Cole ST, Brosch R. 2004. Macro-array and bioinformatic analyses reveal mycobacterial 'core' genes, variation in the ESAT-6 gene family and new phylogenetic markers for the *Mycobacterium tuberculosis* complex. *Microbiology* **150:** (2) 483.
Tamaoki M, Nakajima N, Kubo A, Aono M, Matsuyama T, Saji H. 2003. Transcriptome analysis of $O_3$-exposed *Arabidopsis* reveals that multiple signal pathways act mutually antagonistically to induce gene expression. *Plant Mol Biol* **53:** (4) 443.

### 7 Pharmacogenomics

Adib TR, Henderson S, Perrett C, Hewitt D, Bourmpoulia D, Ledermann J, Boshoff C. 2004. Predicting biomarkers for ovarian cancer using gene-expression microarrays. *Br J Cancer* **90:** (3) 686.
Adachi T, Ono Y, Koh KB, Takashima K, Tainaka H, Matsuno Y, Nakagawa S, Todaka E, Sakurai K, Fukata H *et al*. 2004. Long-term alteration of gene expression without morphological change in testis after neonatal exposure to genistein in mice: Toxicogenomic analysis using cDNA microarray. *Food Chem Toxicol* **42:** (3) 445.
Albertson DN, Pruetz B, Schmidt CJ, Kuhn DM, Kapatos G, Bannon MJ. 2004. Gene expression profile of the nucleus accumbens of human cocaine abusers: Evidence for dysregulation of myelin. *J Neurochem* **88:** (5) 1211.
Alexe G, Alexe S, Liotta LA, Petricoin E, Reiss M, Hammer PL. 2004. Ovarian cancer detection by logical analysis of proteomic data. *Proteomics* **4:** (3) 766.
Alfonso P, Catala M, Rico-Morales ML, Durante-Rodriguez G, Moro-Rodriguez E, Fernandez-Garcia H, Escribano JM, Alvarez-Fernandez E, Garcia-Poblete E. 2004. Proteomic analysis of lung biopsies: Differential protein expression profile between peritumoral and tumoral tissue. *Proteomics* **4:** (2) 442.
Amatschek S, Koenig U, Auer H, Steinlein P, Pacher M, Gruenfelder A, Dekan G, Vogl S, Kubista E, Heider KH *et al*. 2004. Tissue-wide expression profiling using cDNA subtraction and microarrays to identify tumor-specific genes. *Cancer Res* **64:** (3) 844.
An J, Sun JY, Yuan Q, Tian HY, Qiu WL, Guo W, Zhao FK. 2004. Proteomics analysis of differentially expressed metastasis-associated proteins in adenoid cystic carcinoma cell lines of human salivary gland. *Oral Oncol* **40:** (4) 400.
Berman DM, Shih LM, Burke LA, Veenstra TD, Zhao YM, Conrads TP, Kwon SW, Hoang V, Yu LR, Zhou M *et al*. 2004. Profiling the activity of G proteins in patient-derived tissues by rapid affinity-capture of signal transduction proteins (GRASP). *Proteomics* **4:** (3) 812.
Bertucci F, Salas S, Eysteries S, Nasser V, Finetti P, Ginestier C, Charafe-Jauffret E, Loriod B, Bachelart L, Montfort J *et al*. 2004. Gene expression profiling of colon cancer by DNA microarrays and correlation with histoclinical parameters. *Oncogene* **23:** (7) 1377.
Bisca A, D'Ambrosio C, Scaloni A, Puglisi F, Aprile G, Piga A, Zuiani C, Bazzocchi M, Di Loreto C, Paron I *et al*. 2004. Proteomic evaluation of core biopsy specimens from breast lesions. *Cancer Lett* **204:** (1) 79.
Carlson KA, Ciborowski P, Schellpeper CN, Biskup TM, Shen RF, Luo XG, Destache CJ, Gendelman HE. 2004. Proteomic fingerprinting of HIV-1-infected human monocyte-derived macrophages: A preliminary report. *J Neuroimmunol* **147:** (1-2) 35.
Comunale MA, Mattu TS, Lowman MA, Evans AA, London WT, Semmes OJ, Ward M, Drake R, Romano PR, Steel LF *et al*. 2004. Comparative proteomic analysis of de-*N*-glycosylated serum from hepatitis B carriers reveals polypeptides that correlate with disease status. *Proteomics* **4:** (3) 826.
Dahlberg PS, Ferrin LF, Grindle SM, Nelson CM, Hoang CD, Jacobson B. 2004. Gene expression profiles in esophageal adenocarcinoma. *Ann Thorac Surg* **77:** (3) 1008.
De Souza AI, McGregor E, Dunn MJ, Rose ML. 2004. Preparation of human heart for laser microdissection and proteomics. *Proteomics* **4:** (3) 578.
Deaciuc IV, Doherty DE, Burikhanov R, Lee EY, Stromberg AJ, Peng XJ, De Villiers WJS. 2004. Large-scale gene profiling of the liver in a mouse model of chronic, intragastric ethanol infusion. *J Hepatol* **40:** (2) 219.
Diamandis EP. 2004. Analysis of serum proteomic patterns for early cancer diagnosis: Drawing attention to potential problems (Commentary). *J Nat Cancer Inst* **96:** (5) 353.
Dorsam ST, Ferrell CM, Dorsam GP, Derynck MK, Vijapurkar U, Khodabakhsh D, Pau B, Bernstein H, Haqq CM, Largman C *et al*. 2004. The transcriptome of the leukemogenic homeoprotein HOXA9 in human hematopoietic cells. *Blood* **103:** (5) 1676.
Fontana S, Pucci-Minafra I, Becchi M, Freyria AM, Minafra S. 2004. Effect of collagen substrates on proteomic modulation of breast cancer cells. *Proteomics* **4:** (3) 849.
Friedman DB, Hill S, Keller JW, Merchant NB, Levy SE, Coffey RJ, Caprioli RM. 2004. Proteome analysis of human colon cancer by two-dimensional difference gel electrophoresis and mass spectrometry. *Proteomics* **4:** (3) 793.
Gao JJ, Diesl V, Wittmann T, Morrison DC, Ryan JL, Vogel SN, Follettie MT. 2003. Bacterial LPS and CpG DNA differentially induce gene expression profiles in mouse macrophages. *J Endotoxin Res* **9:** (4) 237.
Giorgianni F, Beranova-Giorgianni S, Desiderio DM. 2004. Identification and characterization of phosphorylated proteins in the human pituitary. *Proteomics* **4:** (3) 587.
Gruvberger-Saal SK, Eden P, Ringner M, Baldetorp B, Chebil G, Borg A, Ferno M, Peterson C, Meltzer PS. 2004. Predicting continuous values of prognostic markers in breast cancer from microarray gene expression profiles. *Mol Cancer Ther* **3:** (2) 161.
Hamler RL, Zhu K, Buchanani NS, Kreunin P, Kachman MT, Miller FR, Lubman DM. 2004. A two-dimensional liquid-phase separation method coupled with mass spectrometry for proteomic studies of breast cancer and biomarker identification. *Proteomics* **4:** (3) 562.
Hao XS, Sun BC, Hu LM, Lahdesmaki H, Dunmire V, Feng YM, Zhang SW, Wang HM, Wu CL, Wang H *et al*. 2004. Differential gene and protein expression in primary breast malignancies and their lymph node metastases as revealed by combined cDNA microarray and tissue microarray analysis. *Cancer* **100:** (6) 1110.
Hashimoto T, Lawton MT, Wen G, Yang GY, Chaly T, Stewart CL, Dressman HK, Barbaro NM, Marchuk DA, Young WL. 2004. Gene microarray analysis of human brain arteriovenous malformations. *Neurosurgery* **54:** (2) 410.
Hernandez R, Nombela C, Diez-Orejas R, Gil C. 2004. Two-dimensional reference map of *Candida albicans* hyphal forms. *Proteomics* **4:** (2) 374.
Herzog A, Kuntz S, Daniel H, Wenzel U. 2004. Identification of biomarkers for the initiation of apoptosis in human preneoplastic colonocytes by proteome analysis. *Int J Cancer* **109:** (2) 220.
Hu LP, Evers S, Lu ZH, Shen Y, Chen J. 2004. Two-dimensional protein database of human pancreas. *Electrophoresis* **25:** (3) 512.
Jacobs JM, Mottaz HM, Yu LR, Anderson DJ, Moore RJ, Chen WNU, Auberry KJ, Strittmatter EF, Monroe ME, Thrall BD *et al*. 2004. Multidimensional proteome analysis of human mammary epithelial cells. *J Proteome Res* **3:** (1) 68.
Jones MH, Virtanen C, Honjoh D, Miyoshi T, Satoh Y, Okumura S, Nakagawa K, Nomura H, Ishikawa Y. 2004. Two prognostically significant subtypes of high-grade lung neuroendocrine tumours independent of small-cell and large-cell neuroendocrine carcinomas identified by gene expression profiles. *Lancet* **363:** (9411) 775.
Joo WA, Suli D, Lee DY, Lee EN, Kim CW. 2004. Proteomic analysis of plasma proteins of workers exposed to benzene. *Mutat Res* **558:** (1-2) 35.
Kampa D, Cheng J, Kapranov P, Yamanaka M, Brubaker S, Cawley S, Drenkow J, Piccolboni A, Bekiranov S, Helt G *et al*. 2004. Novel RNAs identified from an in-depth analysis of the transcriptome of human chromosomes 21 and 22. *Genome Res* **14:** (3) 331.
Kim Y, Jang JH, Ku Y, Koak JY, Chang IT, Kim HE, Lee JB, Heo SJ. 2004. Microarray-based expression analysis of human osteoblast-like cell response to anodized titanium surface. *Biotechnol Lett* **26:** (5) 399.
Kleno TG, Leonardsen LR, Kjeldal HO, Laursen SM, Jensen ON, Baunsgaard D. 2004. Mechanisms of hydrazine toxicity in rat liver investigated by proteomics and multivariate data analysis. *Proteomics* **4:** (3) 868.
Korenberg MJ. 2004. On predicting medulloblastoma metastasis by gene expression profiling. *J Proteome Res* **3:** (1) 91.
Lai LP, Lin JL, Lin CS, Yeh HM, Tsay YG, Lee CF, Lee HH, Chang

Copyright © 2004 John Wiley & Sons, Ltd. 

ZF, Hwang JJ, Su MJ *et al*. 2004. Functional genomic study on atrial fibrillation using cDNA microarray and two-dimensional protein electrophoresis techniques and identification of the myosin regulatory light chain isoform reprogramming in atrial fibrillation. *J Cardiovasc Electrophysiol* **15:** (2) 214.

Leak LV, Liotta LA, Krutzsch H, Jones M, Fusaroa VA, Ross SJ, Zhaos YM, Petricoin EF. 2004. Proteomic analysis of lymph. *Proteomics* **4:** (3) 753.

Leszczynski D, Nylund R, Joenvaara S, Reivinen J. 2004. Applicability of discovery science approach to determine biological effects of mobile phone radiation. *Proteomics* **4:** (2) 426.

Maxwell SA, Davis GE. 2004. Gene expression profiling of p53-sensitive and -resistant tumor cells using DNA microarray. *Apoptosis* **9:** (2) 171.

Pang ZJ, Xing FQ. 2004. DNA microarrays detect the expression of apoptosis-related genes in preeclamptic placentas. *J Perinat Med* **32:** (1) 25.

Park JY, Seong JK, Paik YK. 2004. Proteomic analysis of diet-induced hypercholesterolemic mice. *Proteomics* **4:** (2) 514.

Pass HI, Liu ZD, Wali A, Bueno R, Land S, Lott D, Siddiq F, Lonardo F, Carbone M, Draghici S. 2004. Gene expression profiles predict survival and progression of pleural mesothelioma. *Clin Cancer Res* **10:** (3) 849.

Quero C, Colome N, Prieto MR, Carrascal M, Posada M, Gelpi E, Abian J. 2004. Determination of protein markers in human serum: Analysis of protein expression in toxic oil syndrome studies. *Proteomics* **4:** (2) 303.

Roberts K, Bhatia K, Stanton P, Lord R. 2004. Proteomic analysis of selected prognostic factors of breast cancer. *Proteomics* **4:** (3) 784.

Sato N, Fukushima N, Matsubayashi H, Goggins M. 2004. Identification of maspin and S100P as novel hypomethylation targets in pancreatic cancer using global gene expression profiling. *Oncogene* **23:** (8) 1531.

Schwartz SA, Weil RJ, Johnson MD, Toms SA, Caprioli RM. 2004. Protein profiling in brain tumors using mass spectrometry: Feasibility of a new technique for the analysis of protein expression. *Clin Cancer Res* **10:** (3) 981.

Sibille E, Arango V, Galfalvy HC, Pavlidis P, Erraji-Benchekroun L, Ellis SP, Mann JJ. 2004. Gene expression profiling of depression and suicide in human prefrontal cortex. *Neuropsychopharmacology* **29:** (2) 351.

Simpson DM, Beynon RJ, Robertson DHL, Loughran MJ, Haywood S. 2004. Copper-associated liver disease: A proteomics study of copper challenge in a sheep model. *Proteomics* **4:** (2) 524.

Treadwell JA, Singh SM. 2004. Microarray analysis of mouse brain gene expression following acute ethanol treatment. *Neurochem Res* **29:** (2) 357.

Tumour Anal Best Pract Working Grp. 2004. Guidelines - Expression profiling - Best practices for data generation and interpretation in clinical trials. *Nat Rev Genet* **5:** (3) 229.

Van Dekken H, Paris PL, Albertson DG, Alers JC, Andaya A, Kowbel D, Van der Kwast TH, Pinkel D, Schroder FH, Vissers KJ *et al*. 2004. Evaluation of genetic patterns in different tumor areas of intermediate-grade prostatic adenocarcinomas by high-resolution genomic array analysis. *Genes Chromosomes Cancer* **39:** (3) 249.

Vorum H, Ostergaard M, Hensechke P, Enghild JJ, Riazati M, Rice GE. 2004. Proteomic analysis of hyperoxia-induced responses in the human choriocarcinoma cell line JEG-3. *Proteomics* **4:** (3) 861.

Vozenin-Brotons MC, Milliat F, Linard C, Strup C, Francois A, Sabourin JC, Lasser P, Lusinchi A, Deutsch E, Girinsky T *et al*. 2004. Gene expression profile in human late radiation enteritis obtained by high-density cDNA array hybridization. *Radiat Res* **161:** (3) 299.

Watanabe H, Hamada H, Yamada N, Sohda S, Yamakawa-Kobayashi K, Yoshikawa H, Arinami T. 2004. Proteome analysis reveals elevated serum levels of clusterin in patients with preeclampsia. *Proteomics* **4:** (2) 537.

Weeraratna AT, Becker D, Carr KM, Duray PH, Rosenblatt KP, Yang S, Chen YD, Bittner M, Strausberg RL, Riggins GJ *et al*. 2004. Generation and analysis of melanoma SAGE libraries: SAGE advice on the melanoma transcriptome. *Oncogene* **23:** (12) 2264.

Welle S, Brooks AI, Delehanty JM, Needler N, Bhatt K, Shah B, Thornton CA. 2004. Skeletal muscle gene expression profiles in 20-29 year old and 65-71 year old women. *Exp Gerontol* **39:** (3) 369.

Wenner BR, Lovell MA, Lynn BC. 2004. Proteomic analysis of human ventricular cerebrospinal fluid from neurologically normal, elderly subjects using two-dimensional LC-MS/MS. *J Proteome Res* **3:** (1) 97.

Wong ML, O'Kirwan F, Hannestad JP, Irizarry KJL, Elashoff D, Licinio J. 2004. St John's wort and imipramine-induced gene expression profiles identify cellular functions relevant to antidepressant action and novel pharmacogenetic candidates for the phenotype of antidepressant treatment response. *Mol Psychiatry* **9:** (3) 237.

Ying WT, Hao YW, Zhang YJ, Peng WM, Qin E, Cai Y, Wei KH, Wang J, Chang GH, Sun W *et al*. 2004. Proteomic analysis on structural proteins of severe acute respiratory syndrome coronavirus. *Proteomics* **4:** (2) 492.

### 8 EST, cDNA and other clone resources

Brown AC, Kai K, May ME, Brown DC, Roopenian DC. 2004. ExQuest, a novel method for displaying quantitative gene expression from ESTs. *Genomics* **83:** (3) 528.

Close TJ, Wanamaker SI, Caldo RA, Turner SM, Ashlock DA, Dickerson JA, Wing RA, Muehlbauer GJ, Kleinhofs A, Wise RP. 2004. A new resource for cereal genomics: 22K barley GeneChip comes of age. *Plant Physiol* **134:** (3) 960.

Dejardin A, Leple JC, Lesage-Descauses MC, Costa G, Pilate G. 2004. Expressed sequence tags from poplar wood tissues - A comparative analysis from multiple libraries. *Plant Biol* **6:** (1) 55.

Dhanaraj AL, Slovin JP, Rowland LJ. 2004. Analysis of gene expression associated with cold acclimation in blueberry floral buds using expressed sequence tags. *Plant Sci* **166:** (4) 863.

Kodzius R, Matsumura Y, Kasukawa T, Shimokawa K, Fukuda S, Shiraki T, Nakamura M, Arakawa T, Sasaki D, Kawai J *et al*. 2004. Absolute expression values for mouse transcripts: Re-annotation of the READ expression database by the use of CAGE and EST sequence tags. *FEBS Lett* **559:** (1-3) 22.

Kuromori T, Hirayama T, Kiyosue Y, Takabe H, Mizukado S, Sakurai T, Akiyama K, Kamiya A, Ito T, Shinozaki K. 2004. A collection of 11 800 single-copy *Ds* transposon insertion lines in *Arabidopsis*. *Plant J* **37:** (6) 897.

Lee HS, Wang JL, Tian L, Jiang HM, Black MA, Madlung A, Watson B, Lukens L, Pires JC, Wang JJ *et al*. 2004. Sensitivity of 70-mer oligonucleotides and cDNAs for microarray analysis of gene expression in *Arabidopsis* and its related species. *Plant Biotechnol J* **2:** (1) 45.

Nugent KG, Choffe K, Saville BJ. 2004. Gene expression during *Ustilago maydis* diploid filamentous growth: EST library creation and analyses. *Fungal Genet Biol* **41:** (3) 349.

Park JS, Kim JB, Hahn BS, Kim KH, Ha SH, Kim JB, Kim YH. 2004. EST analysis of genes involved in secondary metabolism in *Camellia sinensis* (tea), using suppression subtractive hybridization. *Plant Sci* **166:** (4) 953.

Porcel BM, Delfour O, Castelli V, De Berardinis V, Friedlander L, Cruaud C, Ureta-Vidal A, Scarpelli C, Wincker P, Schachter V *et al*. 2004. Numerous novel annotations of the human genome sequence supported by a 5′-end-enriched cDNA collection. *Genome Res* **14:** (3) 463.

Rise ML, Von Schalburg KR, Brown GD, Mawer MA, Devlin RH, Kuipers N, Busby M, Beetz-Sargent M, Alberto R, Gibbs AR *et al*. 2004. Development and application of a salmonid EST database and cDNA microarray: Data mining and interspecific hybridization characteristics. *Genome Res* **14:** (3) 478.

Xing Y, Resch A, Lee C. 2004. The multiassembly problem: Reconstructing multiple transcript isoforms from EST fragment mixtures. *Genome Res* **14:** (3) 426.

### 9 Functional genomics

Aouida M, Page N, Leduc A, Peter M, Ramotar D. 2004. A genome-wide screen in *Saccharomyces cerevisiae* reveals altered transport as a mechanism of resistance to the anticancer drug bleomycin. *Cancer Res* **64:** (3) 1102.

Brumbley SM, Petrasovits LA, Murphy RM, Nagel RJ, Candy JM, Hermann SR. 2004. Establishment of a functional genomics platform for *Leifsonia xyli* subsp *xyli*. *Mol Plant Microbe Interact* **17:** (2) 175.

Christophides GK, Vlachou D, Kafatos FC. 2004. Comparative and functional genomics of the innate immune system in the malaria vector *Anopheles gambiae*. *Immunol Rev* **198:** (1) 127.

Copyright © 2004 John Wiley & Sons, Ltd. 

Huang QH, Raya A, DeJesus P, Chao SH, Quon KC, Caldwell JS, Chanda SK, Izpisua-Belmonte JC, Schultz PG. 2004. Identification of p53 regulators by genome-wide functional analysis. *Proc Natl Acad Sci U S A* **101:** (10) 3456.
Kohli A, Prynne MQ, Miro B, Pereira A, Twyman RM, Capell T, Christou P. 2004. Dedifferentiation-mediated changes in transposition behavior make the Activator transposon an ideal tool for functional genomics in rice. *Mol Breeding* **13:** (2) 177.
Lu BF, Zagouras P, Fischer JE, Lu JF, Li BY, Flavell RA. 2004. Kinetic analysis of genomewide gene expression reveals molecule circuitries that control T cell activation and Th1/2 differentiation. *Proc Natl Acad Sci U S A* **101:** (9) 3023.
Marston AL, Tham WH, Shah H, Amon A. 2004. A genome-wide screen identifies genes required for centromeric cohesion. *Science* **303:** (5662) 1367.
Tanay A, Sharan R, Kupiec M, Shamir R. 2004. Revealing modularity and organization in the yeast molecular network by integrated analysis of highly heterogeneous genomewide data. *Proc Natl Acad Sci U S A* **101:** (9) 2981.
Van der Kaaij H, Desiere F, Mollet B, Germond JE. 2004. L-Alanine auxotrophy of *Lactobacillus johnsonii* as demonstrated by physiological, genomic, and gene complementation approaches. *Appl Environ Microbiol* **70:** (3) 1869.
Yazaki J, Kishimoto N, Nagata Y, Ishikawa M, Fujii F, Hashimoto A, Shimbo K, Shimatani Z, Kojma K, Suzuki K *et al*. 2003. Genomics approach to abscisic acid- and gibberellin-responsive genes in rice. *DNA Res* **10:** (6) 249.
Yu JW, Mendrola JM, Audhya A, Singh S, Keleti D, DeWald DB, Murray D, Emr SD, Lemmon MA. 2004. Genome-wide analysis of membrane targeting by *S. cerevisiae* pleckstrin homology domains. *Mol Cell* **13:** (5) 677.

### 10 Transcriptomics

Bernstein JA, Lin PH, Cohen SN, Lin-Chao S. 2004. Global analysis of *Escherichia coli* RNA degradosome function using DNA microarrays. *Proc Natl Acad Sci U S A* **101:** (9) 2758.
Bignell GR, Huang J, Greshock J, Watt S, Butler A, West S, Grigorova M, Jones KW, Wei W, Stratton MR *et al*. 2004. High-resolution analysis of DNA copy number using oligonucleotide microarrays. *Genome Res* **14:** (2) 287.
Frederiksen KS, Wulff EM, Sauerberg P, Mogensen JP, Jeppesen L, Fleckner J. 2004. Prediction of PPAR-α ligand-mediated physiological changes using gene expression profiles. *J Lipid Res* **45:** (3) 592.
Gowda M, Jantasuriyarat C, Dean RA, Wang GL. 2004. Robust-LongSAGE (RL-SAGE): A substantially improved LongSAGE method for gene discovery and transcriptome analysis. *Plant Physiol* **134:** (3) 890.
Hansen EH, Schembri MA, Klemm P, Schafer T, Molin S, Gram L. 2004. Elucidation of the antibacterial mechanism of the *Curvularia* haloperoxidase system by DNA microarray profiling. *Appl Environ Microbiol* **70:** (3) 1749.
Hennig L, Menges M, Murray JAH, Gruissem W. 2003. *Arabidopsis* transcript profiling on Affymetrix GeneChip arrays. *Plant Mol Biol* **53:** (4) 457.
Kaiser K, Matuschewski K, Camargo N, Ross J, Kappe SHI. 2004. Differential transcriptome profiling identifies *Plasmodium* genes encoding pre-erythrocytic stage-specific proteins. *Mol Microbiol* **51:** (5) 1221.
Ko JH, Chow KS, Han KH. 2003. Transcriptome analysis reveals novel features of the molecular events occurring in the laticifers of *Hevea brasiliensis* (para rubber tree). *Plant Mol Biol* **53:** (4) 479.
Leonhardt N, Kwak JM, Robert N, Waner D, Leonhardt G, Schroeder JI. 2004. Microarray expression analyses of *Arabidopsis* guard cells and isolation of a recessive abscisic acid hypersensitive protein phosphatase 2C mutant. *Plant Cell* **16:** (3) 596.
Lesur I, Campbell JL. 2004. The transcriptome of prematurely aging yeast cells is similar to that of telomerase-deficient cells. *Mol Biol Cell* **15:** (3) 1297.
Lin WH, Ye R, Ma H, Xu ZH, Xue HW. 2004. DNA chip-based expression profile analysis indicates involvement of the phosphatidylinositol signaling pathway in multiple plant responses to hormone and abiotic treatments. *Cell Res* **14:** (1) 34.
Lister R, Chew O, Lee MN, Heazlewood JL, Clifton R, Parker KL, Millar AH, Whelan J. 2004. A transcriptomic and proteomic characterization of the *Arabidopsis* mitochondrial protein import apparatus and its response to mitochondrial dysfunction. *Plant Physiol* **134:** (2) 777.
Liu XQ, De Wulf P. 2004. Probing the ArcA-P modulon of *Escherichia coli* by whole genome transcriptional analysis and sequence recognition profiling. *J Biol Chem* **279:** (13) 12588.
Menges M, Hennig L, Gruissem W, Murray JAH. 2003. Genome-wide gene expression in an *Arabidopsis* cell suspension. *Plant Mol Biol* **53:** (4) 423.
Mostertz J, Scharf C, Hecker M, Homuth G. 2004. Transcriptome and proteome analysis of *Bacillus subtilis* gene expression in response to superoxide and peroxide stress. *Microbiology* **150:** (2) 497.
Perez-Enciso M. 2004. *In silico* study of transcriptome genetic variation in outbred populations. *Genetics* **166:** (1) 547.
Ranz JM, Namgyal K, Gibson G, Hartl DL. 2004. Anomalies in the expression profile of interspecific hybrids of *Drosophila melanogaster* and *Drosophila simulans*. *Genome Res* **14:** (3) 373.
Roh JH, Smith WE, Kaplan S. 2004. Effects of oxygen and light intensity on transcriptome expression in *Rhodobacter sphaeroides* 2.4.1 - Redox active gene expression profile. *J Biol Chem* **279:** (10) 9146.
Vincentz M, Cara FAA, Okura VK, Da Silva FR, Pedrosa GL, Hemerly AS, Capella AN, Marins M, Ferreira PC, Franca SC *et al*. 2004. Evaluation of monocot and eudicot divergence using the sugarcane transcriptome. *Plant Physiol* **134:** (3) 951.
Watahiki J, Yamaguchi T, Irie T, Nakano H, Maki K, Tachikawa T. 2004. Gene expression profiling of mouse condylar cartilage during mastication by means of laser microdissection and cDNA array. *J Dent Res* **83:** (3) 245.

### 11 Proteomics

Agudo D, Mendoza MT, Castanares C, Nombela C, Rotger R. 2004. A proteomic approach to study *Salmonella typhi* periplasmic proteins altered by a lack of the DsbA thiol: disulfide isomerase. *Proteomics* **4:** (2) 355.
Akimoto H, Wu C, Kinumi T, Ohmiya Y. 2004. Biological rhythmicity in expressed proteins of the marine dinoflagellate *Lingulodinium polyedrum* demonstrated by chronological proteomics. *Biochem Biophys Res Commun* **315:** (2) 306.
Bahrman N, Le Gouis J, Negroni L, Amilhat L, Leroy P, Laine AL, Jaminon O. 2004. Differential protein expression assessed by two-dimensional gel electrophoresis for two wheat varieties grown at four nitrogen levels. *Proteomics* **4:** (3) 709.
Baik SC, Kim KM, Song SM, Kim DS, Jun JS, Lee SG, Song JY, Park JU, Kang HL, Lee WK *et al*. 2004. Proteomic analysis of the sarcosine-insoluble outer membrane fraction of *Helicobacter pylori* strain 26695. *J Bacteriol* **186:** (4) 949.
Bak-Jensen KS, Laugesen S, Roepstorff P, Svensson B. 2004. Two-dimensional gel electrophoresis pattern (pH 6-11) and identification of water-soluble barley seed and malt proteins by mass spectrometry. *Proteomics* **4:** (3) 728.
Balmer Y, Koller A, Del Val G, Schurmann P, Buchanan BB. 2004. Proteomics uncovers proteins interacting electrostatically with thioredoxin in chloroplasts. *Photosynth Res* **79:** (3) 275.
Becker F, Murthi K, Smith C, Come J, Costa-Roldan N, Kaufmann C, Hanke U, Degenhart C, Baumann S, Wallner W *et al*. 2004. A three-hybrid approach to scanning the proteome for targets of small molecule kinase inhibitors. *Chem Biol* **11:** (2) 211.
Besant PG, Lasker MV, Bui CD, Tan E, Attwood PV, Turck CW. 2004. Proteomics approach to identifying ATP-covalently modified proteins. *J Proteome Res* **3:** (1) 120.
Bock C, Coleman M, Collins B, Davis J, Foulds G, Gold L, Greef C, Heil J, Heilig JS, Hicke B *et al*. 2004. Photoaptamer arrays applied to multiplexed proteomic analysis. *Proteomics* **4:** (3) 609.
Brajenovic M, Joberty G, Kuster B, Bouwmeester T, Drewes G. 2004. Comprehensive proteomic analysis of human Par protein complexes reveals an interconnected protein network. *J Biol Chem* **279:** (13) 12804.
Campo S, Carrascal M, Coca M, Abian J, San Segundo B. 2004. The defense response of germinating maize embryos against fungal infection: A proteomics approach. *Proteomics* **4:** (2) 383.
Castegna A, Thongboonkerd V, Klein J, Lynn BC, Wang YL, Osaka H, Wada K, Butterfield DA. 2004. Proteomic analysis of brain proteins in the gracile axonal dystrophy (*gad*) mouse, a syndrome that emanates

Copyright © 2004 John Wiley & Sons, Ltd. 

from dysfunctional ubiquitin carboxyl-terminal hydrolase-L-1, reveals oxidation of key proteins. *J Neurochem* **88:** (6) 1540.
Catalano CM, Lane WS, Sherrier DJ. 2004. Biochemical characterization of symbiosome membrane proteins from *Medicago truncatula* root nodules. *Electrophoresis* **25:** (3) 519.
Chitlaru T, Ariel N, Zvi A, Lion M, Velan B, Shaffermann A, Elhanany E. 2004. Identification of chromosomally encoded membranal polypeptides of *Bacillus anthracis* by a proteomic analysis: Prevalence of proteins containing S-layer homology domains. *Proteomics* **4:** (3) 677.
Corton M, Villuendas G, Botella JI, San Millan JL, Escobar-Morreale HF, Peral B. 2004. Improved resolution of the human adipose tissue proteome at alkaline and wide range pH by the addition of hydroxyethyl disulfide. *Proteomics* **4:** (2) 438.
Crettaz D, Sensebe L, Vu DH, Schneider P, Depasse F, Bienvenut WV, Quadroni M, Tissot JD. 2004. Proteomics of methylene blue photo-treated plasma before and after removal of the dye by an absorbent filter. *Proteomics* **4:** (3) 881.
Dumas-Gaudot E, Amiour N, Weidmann S, Bestel-Correl G, Valot B, Lenogue S, Gianinazzi-Pearson V, Gianinazzi S. 2004. A technical trick for studying proteomics in parallel to transcriptomics in symbiotic root-fungus interactions. *Proteomics* **4:** (2) 451.
Garcia A, Prabhakar S, Brock CJ, Pearce AC, Dwek RA, Watson SP, Hebestreit HF, Zitzmann N. 2004. Extensive analysis of the human platelet proteome by two-dimensional gel electrophoresis and mass spectrometry. *Proteomics* **4:** (3) 656.
Gonzalez-Barderas M, Gallego-Delgado J, Mas S, Duran MC, Lazaro A, Hernandez-Merida S, Egido J, Vivanco F. 2004. Isolation of circulating human monocytes with high purity for proteomic analysis. *Proteomics* **4:** (2) 432.
Gonzalez-Camacho F, Medina FJ. 2004. Identification of specific plant nucleolar phosphoproteins in a functional proteomic analysis. *Proteomics* **4:** (2) 407.
Grinyer J, McKay M, Nevalainen H, Herbert BR. 2004. Fungal proteomics: Initial mapping of biological control strain *Trichoderma harzianum*. *Curr Genet* **45:** (3) 163.
Grinyer J, McKay M, Herbert B, Nevalainen H. 2004. Fungal proteomics: Mapping the mitochondrial proteins of a *Trichoderma harzianum* strain applied for biological control. *Curr Genet* **45:** (3) 170.
Grunberg K, Muller EC, Otto A, Reszka R, Linder D, Kube M, Reinhardt R, Schuler D. 2004. Biochemical and proteomic analysis of the magnetosome membrane in *Magnetospitillum gryphiswaldense*. *Appl Environ Microbiol* **70:** (2) 1040.
Hernandez G, Lalioti V, Vandekerckhove J, Sierra JM, Santaren JF. 2004. Identification and characterization of the expression of the translation initiation factor 4A (eIF4A) from *Drosophila melanogaster*. *Proteomics* **4:** (2) 316.
Hickman HD, Luis AD, Buchli R, Few SR, Sathiamurthy M, Van Gundy RS, Giberson CF, Hildebrand WH. 2004. Toward a definition of self: Proteomic evaluation of the class I peptide repertoire. *J Immunol* **172:** (5) 2944.
Holland JW, Deeth HC, Alewood PF. 2004. Proteomic analysis of κ-casein micro-heterogeneity. *Proteomics* **4:** (3) 743.
Hu H, Columbus J, Zhang Y, Wu DY, Lian LB, Yang S, Goodwin J, Luczak C, Carter M, Chen L *et al*. 2004. A map of WW domain family interactions. *Proteomics* **4:** (3) 643.
Jin JH, Yang J, Gao ZH, Yu YN. 2004. Proteomic analysis of cellular responses to low concentration *N*-methyl-*N*-nitro-*N*-nitrosoguanidine in human amnion FL cells. *Environ Mol Mutagen* **43:** (2) 93.
Juarez P, Sanz L, Calvete JJ. 2004. Snake venomics: Characterization of protein families in *Sistrurus barbouri* venom by cysteine mapping, N-terminal sequencing, and tandem mass spectrometry analysis. *Proteomics* **4:** (2) 327.
Kleffmann T, Russenberger D, Von Zychlinski A, Christopher W, Sjolander K, Gruissem W, Baginsky S. 2004. The *Arabidopsis thaliana* chloroplast proteome reveals pathway abundance and novel protein functions. *Curr Biol* **14:** (5) 354.
Laukens K, Deckers P, Esmans E, Van Onckelen H, Witters E. 2004. Construction of a two-dimensional gel electrophoresis protein database for the *Nicotiana tabacum* cv. Bright Yellow-2 cell suspension culture. *Proteomics* **4:** (3) 720.
Lee K, Lee J, Kim Y, Bae D, Kang KY, Yoon SC, Lim D. 2004. Defining the plant disulfide proteome. *Electrophoresis* **25:** (3) 532.
Lee KA, Shim JH, Kho CW, Park SG, Park BC, Kim JW, Lim JS, Choe YK, Paik SG, Yoon DY. 2004. Protein profiling and identification of modulators regulated by the E7 oncogene in the C33A cell line by proteomics and genomics. *Proteomics* **4:** (3) 839.
Lindahl M, Florencio FJ. 2004. Systematic screening of reactive cysteine proteomes. *Proteomics* **4:** (2) 448.
Majoul T, Bancel E, Triboi E, Ben Hamida J, Branlard G. 2004. Proteomic analysis of the effect of heat stress on hexaploid wheat grain: Characterization of heat-responsive proteins from non-prolamins fraction. *Proteomics* **4:** (2) 505.
Martinez I, Friis TJ. 2004. Application of proteome analysis to seafood authentication. *Proteomics* **4:** (2) 347.
Medina ML, Kiernan UA, Francisco WA. 2004. Proteomic analysis of rutin-induced secreted proteins from *Aspergillus flavus*. *Fungal Genet Biol* **41:** (3) 327.
Navarro-Lerida I, Moreno MM, Roncal F, Gavilanes F, Albar JP, Rodriguez-Crespo I. 2004. Proteomic identification of brain proteins that interact with dynein light chain LC8. *Proteomics* **4:** (2) 339.
Orchard S, Hermjakob H, Julian RK, Runte K, Sherman D, Wojcik J, Zhu WM, Apweiler R. 2004. Common interchange standards for proteomics data: Public availability of tools and schema. *Proteomics* **4:** (2) 490.
Pan Y, Kislinger T, Gramolini AO, Zvaritch E, Kranias EG, MacLennan DH, Emili A. 2004. Identification of biochemical adaptations in hyper- or hypocontractile hearts from phospholamban mutant mice by expression proteomics. *Proc Natl Acad Sci U S A* **101:** (8) 2241.
Paulson L, Martin P, Nilsson CL, Ljung E, Westman-Brinkmalm A, Blennow K, Davidsson P. 2004. Comparative proteome analysis of thalamus in MK-801-treated rats. *Proteomics* **4:** (3) 819.
Perez-Bueno ML, Rahoutei J, Sajnani C, Garcia-Luque I, Baron M. 2004. Proteomic analysis of the oxygen-evolving complex of photosystem II under biotec stress: Studies on *Nicotiana benthamiana* infected with tobamoviruses. *Proteomics* **4:** (2) 418.
Ren DY, Penner NA, Slentz BE, Regnier FE. 2004. Histidine-rich peptide selection and quantification in targeted proteomics. *J Proteome Res* **3:** (1) 37.
Renaut J, Lutts S, Hoffmann L, Hausman JF. 2004. Responses of poplar to chilling temperatures: Proteomic and physiological aspects. *Plant Biol* **6:** (1) 81.
Resch A, Xing Y, Modrek B, Gorlick M, Riley R, Lee C. 2004. Assessing the impact of alternative splicing on domain interactions in the human proteome. *J Proteome Res* **3:** (1) 76.
Roberts MD, Martin NL, Kropinski AM. 2004. The genome and proteome of coliphage T1. *Virology* **318:** (1) 245.
Rose A, Manikantan S, Schraegle SJ, Maloy MA, Stahlberg EA, Meier I. 2004. Genome-wide identification of *Arabidopsis* coiled-coil proteins and establishment of the ARABI-COIL database. *Plant Physiol* **134:** (3) 927.
Schulze WX, Mann M. 2004. A novel proteomic screen for peptide-protein interactions. *J Biol Chem* **279:** (11) 10756.
Sem DS, Bertolaet B, Baker B, Chang E, Costache AD, Coutts S, Dong Q, Hansen M, Hong V, Huang XM *et al*. 2004. Systems-based design of bi-ligand inhibitors of oxidoreductases: Filling the chemical proteomic toolbox. *Chem Biol* **11:** (2) 185.
Shim JH, Danielpour D, Lee C, Kim YS, Bahk YY, Yoo TK. 2004. Proteome profile changes during transdifferentiation of NRP-152 rat prostatic basal epithelial cells. *Mol Cells* **17:** (1) 108.
Vido K, le Bars D, Mistou MY, Anglade P, Gruss A, Gaudu P. 2004. Proteome analyses of heme-dependent respiration in *Lactococcus lactis*: Involvement of the proteolytic system. *J Bacteriol* **186:** (6) 1648.
Vilain S, Cosette P, Zimmerlin I, Dupont JP, Junter GA, Jouenne T. 2004. Biofilm proteome: Homogeneity or versatility? *J Proteome Res* **3:** (1) 132.
Wagner V, Fiedler M, Markert C, Hippler M, Mittag M. 2004. Functional proteomics of circadian expresses proteins from *Chlamydomonas reinhardtii*. *FEBS Lett* **559:** (1-3) 129.
Wang SB, Hu Q, Sommerfeld M, Chen F. 2004. Cell wall proteomics of the green alga *Haematococcus pluvialis* (Chlorophyceae). *Proteomics* **4:** (3) 692.
Zhou B, Yang W, Ji JG, Ru BG. 2004. Differential display proteome analysis of PC-12 cells transiently transfected with metallothionein-3 gene. *J Proteome Res* **3:** (1) 126.

Copyright © 2004 John Wiley & Sons, Ltd. 

### I2 Protein structural genomics

Finley JB, Qiu SH, Luan CH, Luo M. 2004. Structural genomics for *Caenorhabditis elegans*: High throughput protein expression analysis. *Protein Expr Purif* **34:** (1) 49.

Goda N, Tenno T, Takasu H, Hiroaki H, Shirakawa M. 2004. The PRESAT-vector: Asymmetric T-vector for high-throughput screening of soluble protein domains for structural proteomics. *Protein Sci* **13:** (3) 652.

### I3 Metabolomics

Burgard AP, Nikolaev EV, Schilling CH, Maranas CD. 2004. Flux coupling analysis of genome-scale metabolic network reconstructions. *Genome Res* **14:** (2) 301.

Haurie V, Sagliocco F, Boucherie H. 2004. Dissecting regulatory networks by means of two-dimensional gel electrophoresis: Application to the study of the diauxic shift in the yeast *Saccharomyces cerevisiae*. *Proteomics* **4:** (2) 364.

Jonsson N, Gullberg J, Nordstrom A, Kusano M, Kowalczyk M, Sjostrom M, Moritz T. 2004. A strategy for identifying differences in large series of metabolomic samples analyzed by GC/MS. *Anal Chem* **76:** (6) 1738.

Kromer JO, Sorgenfrei O, Klopprogge K, Heinzle E, Wittmann C. 2004. In-depth profiling of lysine-producing *Corynebacterium glutamicum* by combined analysis of the transcriptome, metabolome, and fluxome. *J Bacteriol* **186:** (6) 1769.

Sun N, Ma LG, Pan DY, Zhao HY, Deng XW. 2003. Evaluation of light regulatory potential of Calvin cycle steps based on large-scale gene expression profiling data. *Plant Mol Biol* **53:** (4) 467.

Thimm O, Blasing O, Gibon Y, Nagel A, Meyer S, Kruger P, Selbig J, Muller LA, Rhee SY, Stitt M. 2004. MAPMAN: A user-driven tool to display genomics data sets onto diagrams of metabolic pathways and other biological processes. *Plant J* **37:** (6) 914.

Tyson GW, Chapman J, Hugenholtz P, Allen EE, Ram RJ, Richardson PM, Solovyev VV, Rubin EM, Rokhsar DS, Banfield JF. 2004. Community structure and metabolism through reconstruction of microbial genomes from the environment. *Nature* **428:** (6978) 37.

### I4 Genomic approaches to development

Ahn JI, Lee KH, Shin DM, Shim JW, Lee JS, Chang SY, Lee YS, Brownstein MJ, Lee SH, Lee YS. 2004. Comprehensive transcriptome analysis of differentiation of embryonic stem cells into midbrain and hindbrain neurons. *Dev Biol* **265:** (2) 491.

Brand M, Ranish JA, Kummer NT, Hamilton J, Igarashi K, Francastel C, Chi TH, Crabtree GR, Aebersold R, Groudine M. 2004. Dynamic changes in transcription factor complexes during erythroid differentiation revealed by quantitative proteomics. *Nat Struct Mol Biol* **11:** (1) 73.

Dickmeis T, Plessy C, Rastegar S, Aanstad P, Herwig R, Chalmel F, Fischer N, Strahle U. 2004. Expression profiling and comparative genomics identify a conserved regulatory region controlling midline expression in the zebrafish embryo. *Genome Res* **14:** (2) 228.

Gong L, Puri M, Unlu M, Young M, Robertson K, Viswanathan S, Krishnaswamy A, Dowd SR, Minden JS. 2004. *Drosophila* ventral furrow morphogenesis: A proteomic analysis. *Development* **131:** (3) 643.

Lonosky PM, Zhang XS, Honavar VG, Dobbs DL, Fu A, Rodermel SR. 2004. A proteomic analysis of maize chloroplast biogenesis. *Plant Physiol* **134:** (2) 560.

Prudhomme W, Daley GQ, Zandstra P, Lauffenburger DA. 2004. Multivariate proteomic analysis of murine embryonic stem cell self-renewal *versus* differentiation signaling. *Proc Natl Acad Sci U S A* **101:** (9) 2900.

Togo S, Makino H, Kobayashi T, Morita T, Shimizu T, Kubota T, Ichikawa Y, Ishikawa T, Okazaki Y, Hayashizaki Y *et al*. 2004. Mechanism of liver regeneration after partial hepatectomy using mouse cDNA microarray. *J Hepatol* **40:** (3) 464.

Zolessi FR, Duran R, Engstrom U, Cervenansky C, Hellman U, Arruti C. 2004. Identification of the chicken MARCKS phosphorylation site specific for differentiating neurons as Ser 25 using a monoclonal antibody and mass spectrometry. *J Proteome Res* **3:** (1) 84.

### I5 Technological advances

Baczek T, Bucinski A, Ivanov AR, Kaliszan R. 2004. Artificial neural network analysis for evaluation of peptide MS/MS spectra in proteomics. *Anal Chem* **76:** (6) 1726.

Bunkenborg J, Pilch BJ, Podtelejnikov AV, Wisniewski JR. 2004. Screening for N-glycosylated proteins by liquid chromatography mass spectrometry. *Proteomics* **4:** (2) 454.

Cargile BJ, Bundy JL, Freeman TW, Stephenson JL. 2004. Gel based isoelectric focusing of peptides and the utility of isoelectric point in protein identification. *J Proteome Res* **3:** (1) 112.

Cargile BJ, Talley DL, Stephenson JL. 2004. Immobilized pH gradients as a first dimension in shotgun proteomics and analysis of the accuracy of pI predictability of peptides. *Electrophoresis* **25:** (6) 936.

Everberg H, Sivars U, Emanuelsson C, Persson C, Englund AK, Haneskog L, Lipniunas P, Jornten-Karlsson M, Tjerneld F. 2004. Protein pre-fractionation in detergent-polymer aqueous two-phase systems for facilitated proteomic studies of membrane proteins. *J Chromatogr A* **1029:** (1-2) 113.

Graber A, Juhasz PS, Khainovski N, Parker KC, Patterson DH, Martin SA. 2004. Result-driven strategies for protein identification and quantitation - A way to optimize experimental design and derive reliable results. *Proteomics* **4:** (2) 474.

He F, Emmett MR, Hakansson K, Hendrickson CL, Marshall AG. 2004. Theoretical and experimental prospects for protein identification based solely on accurate mass measurement. *J Proteome Res* **3:** (1) 61.

Hochleitner EO, Sondermann P, Lottspeich F. 2004. Determination of the stoichiometry of protein complexes using liquid chromatography with fluorescence and mass spectrometric detection of fluorescently labeled proteolytic peptides. *Proteomics* **4:** (3) 669.

Klur S, Toy K, Williams MP, Certa U. 2004. Evaluation of procedures for amplification of small-size samples for hybridization on microarrays. *Genomics* **83:** (3) 508.

Lamanda A, Zahn A, Roder D, Langen H. 2004. Improved ruthenium II tris (bathophenantroline disulfonate) staining and destaining protocol for a better signal-to-background ratio and improved baseline resolution. *Proteomics* **4:** (3) 599.

Lokhov PG, Tikhonova OV, Moshkovskii SA, Goufman EI, Serebriakova MV, Maksimov BI, Toropyguine IY, Zgoda VG, Govorun VM, Archakov AI. 2004. Database search post-processing by neural network: Advanced facilities for identification of components in protein mixtures using mass spectrometric peptide mapping. *Proteomics* **4:** (3) 633.

Luche S, Diemer H, Tastet C, Chevallet M, Van Dorsselaer A, Leize-Wagner E, Rabilloud T. 2004. About thiol derivatization and resolution of basic proteins in two-dimensional electrophoresis. *Proteomics* **4:** (3) 551.

Matsuzaki H, Loi H, Dong S, Tsai YY, Fang J, Law J, Di XJ, Liu WM, Yang G, Liu GY *et al*. 2004. Parallel genotyping of over 10,000 SNPs using a one-primer assay on a high-density oligonucleotide array. *Genome Res* **14:** (3) 414.

Miyagishi M, Taira K. 2003. Strategies for generation of an siRNA expression library directed against the human genome. *Oligonucleotides* **13:** (5) 325.

Nedelkov D, Tubbs KA, Niederkofler EE, Kiernan UA, Nelson RW. 2004. High-throughput comprehensive analysis of human plasma proteins: A step toward population proteomics. *Anal Chem* **76:** (6) 1733.

Padliya ND, Wood TD. 2004. A strategy to improve peptide mass fingerprinting matches through the optimization of matrix-assisted laser desorption/ionization matrix selection and formulation. *Proteomics* **4:** (2) 466.

Pan P, Gunawardena HP, Xia Y, McLuckey SA. 2004. Nanoelectrospray ionization of protein mixtures: Solution pH and protein pI. *Anal Chem* **76:** (4) 1165.

Pitteri SJ, Reid GE, McLuckey SA. 2004. Affecting proton mobility in activated peptide and whole protein ions *via* lysine guanidination. *J Proteome Res* **3:** (1) 46.

Reynolds A, Leake D, Boese Q, Scaringe S, Marshall WS, Khvorova A. 2004. Rational siRNA design for RNA interference. *Nat Biotechnol* **22:** (3) 326.

Shen YF, Jacobs JM, Camp DG, Fang RH, Moore RJ, Smith RD, Xiao WZ, Davis RW, Tompkins RG. 2004. Ultra-high-efficiency strong cation exchange LC/RPLC/MS/MS for high dynamic range characterization of the human plasma proteome. *Anal Chem* **76:** (4) 1134.

Copyright © 2004 John Wiley & Sons, Ltd.

Shi Y, Xiang R, Crawford JK, Colangelo CM, Horvath C, Wilkins JA. 2004. A simple solid phase mass tagging approach for quantitative proteomics. *J Proteome Res* **3:** (1) 104.

Szponarski W, Sommerer N, Boyer JC, Rossignol M, Gibrat R. 2004. Large-scale characterization of integral proteins from *Arabidopsis* vacuolar membrane by two-dimensional liquid chromatography. *Proteomics* **4:** (2) 397.

Tabuchi M, Ueda M, Kaji N, Yamasaki Y, Nagasaki Y, Yoshikawa K, Kataoka K, Baba Y. 2004. Nanospheres for DNA separation chips. *Nat Biotechnol* **22:** (3) 337.

Villanueva J, Philip J, Entenberg D, Chaparro CA, Tanwar MK, Holland EC, Tempst P. 2004. Serum peptide profiling by magnetic particle-assisted, automated sample processing and MALDI-TOF mass spectrometry. *Anal Chem* **76:** (6) 1560.

### I6 Bioinformatics

Al-Shahrour F, Diaz-Uriarte R, Dopazo J. 2004. FatiGO: A web tool for finding significant associations of Gene Ontology terms with groups of genes. *Bioinformatics* **20:** (4) 578.

Andreini C, Banci L, Bertini I, Luchinat C, Rosato A. 2004. Bioinformatic comparison of structures and homology-models of matrix metalloproteinases. *J Proteome Res* **3:** (1) 21.

Bastien O, Aude JC, Roy S, Marechal E. 2004. Fundamentals of massive automatic pairwise alignments of protein sequences: Theoretical significance of *Z*-value statistics. *Bioinformatics* **20:** (4) 534.

Blazewicz J, Formanowicz P, Kasprzak M, Markiewicz WT, Swiercz A. 2004. Tabu search algorithm for DNA sequencing by hybridization with isothermic libraries. *Comput Biol Chem* **28:** (1) 11.

Braga-Neto UM, Dougherty ER. 2004. Is cross-validation valid for small-sample microarray classification? *Bioinformatics* **20:** (3) 374.

Carpentier AS, Riva A, Tisseur P, Didier G, Henaut A. 2004. The operons, a criterion to compare the reliability of transcriptome analysis tools: ICA is more reliable than ANOVA, PLS and PCA. *Comput Biol Chem* **28:** (1) 3.

Cavener JD, Cull P, Holloway JL, Hsu TC. 2004. Walking tree heuristics for comparative genomic alignments. *Math Biosci* **188:** 207.

Chamrad DC, Korting G, Stuhler K, Meyer HE, Klose J, Bluggel M. 2004. Evaluation of algorithms for protein identification from sequence databases using mass spectrometry data. *Proteomics* **4:** (3) 619.

Chapman MA, Donaldson IJ, Gilbert J, Grafham D, Rogers J, Green AR, Gottgens B. 2004. Analysis of multiple genomic sequence alignments: A web resource, online tools, and lessons learned from analysis of mammalian SCL loci. *Genome Res* **14:** (2) 313.

Cheung LWK. 2004. Use of runs statistics for pattern recognition in genomic DNA sequences. *J Comput Biol* **11:** (1) 107.

Chu TM, Weir BS, Wolfinger RD. 2004. Comparison of Li-Wong and loglinear mixed models for the statistical analysis of oligonucleotide arrays. *Bioinformatics* **20:** (4) 500.

Clamp M, Cuff J, Searle SM, Barton GJ. 2004. The Jalview Java alignment editor. *Bioinformatics* **20:** (3) 426.

Clerens S, Van den Ende W, Verhaert P, Geenen L, Arckens L. 2004. Sweet Substitute: A software tool for *in silico* fragmentation of peptide-linked N-glycans. *Proteomics* **4:** (3) 629.

Cope LM, Irizarry RA, Jaffee HA, Wu ZJ, Speed TP. 2004. A benchmark for *Affymetrix GeneChip* expression measures. *Bioinformatics* **20:** (3) 323.

Demir E, Babur O, Dogrusoz U, Gursoy A, Ayaz A, Gulesir G, Nisanci G, Cetin-Atalay R. 2004. An ontology for collaborative construction and analysis of cellular pathways. *Bioinformatics* **20:** (3) 349.

Eriksson J, Fenyo D. 2004. Probity: A protein identification algorithm with accurate assignment of the statistical significance of the results. *J Proteome Res* **3:** (1) 32.

Gautier L, Cope L, Bolstad BM, Irizarry RA. 2004. affy - analysis of *Affymetrix GeneChip* data at the probe level. *Bioinformatics* **20:** (3) 307.

Hornshoj H, Stengaard H, Panitz F, Bendixen C. 2004. SEPON, a Selection and Evaluation Pipeline for OligoNucleotides based on ESTs with a non-target $T_m$ algorithm for reducing cross-hybridization in microarray gene expression experiments. *Bioinformatics* **20:** (3) 428.

Huang Y, Zhang L. 2004. Rapid and sensitive dot-matrix methods for genome analysis. *Bioinformatics* **20:** (4) 460.

Inoue Y, Ikeda M, Shimizu J. 2004. Proteome-wide classification and identification of mammalian-type GPCRs by binary topology pattern. *Comput Biol Chem* **28:** (1) 39.

Karpinets TV, Foy BD, Frazier JM. 2004. Tailored gene array databases: Applications in mechanistic toxicology. *Bioinformatics* **20:** (4) 507.

Keightley PD, Johnson T. 2004. MCALIGN: Stochastic alignment of noncoding DNA sequences based on an evolutionary model of sequence evolution. *Genome Res* **14:** (3) 442.

Kowalski J, Drake C, Schwartz RH, Powell J. 2004. Non-parametric, hypothesis-based analysis of microarrays for comparison of several phenotypes. *Bioinformatics* **20:** (3) 364.

Kutalik Z, Inwald J, Gordon SV, Hewinson RG, Butcher P, Hinds J, Cho KH, Wolkenhauer O. 2004. Advanced significance analysis of microarray data based on weighted resampling: A comparative study and application to gene deletions in *Mycobacterium bovis*. *Bioinformatics* **20:** (3) 357.

Lamers S, Beason S, Dunlap L, Compton R, Salemi M. 2004. HIVbase: a PC/Windows-based software offering storage and querying power for locally held HIV-1 genetic, experimental and clinical data. *Bioinformatics* **20:** (3) 436.

Lavorgna G, Sessa L, Guffanti A, Lassandro L, Casari G. 2004. AntiHunter: Searching BLAST output for EST antisense transcripts. *Bioinformatics* **20:** (4) 583.

Lawrence ND, Milo M, Niranjan M, Rashbass P, Soullier S. 2004. Reducing the variability in cDNA microarray image processing by Bayesian inference. *Bioinformatics* **20:** (4) 518.

Lee SG, Hur JU, Kim YS. 2004. A graph-theoretic modeling on GO space for biological interpretation of gene clusters. *Bioinformatics* **20:** (3) 381.

Levenkova N, Gu QJ, Rux JJ. 2004. Gene specific siRNA selector. *Bioinformatics* **20:** (3) 430.

Li LG, Jin RC, Kok PL, Wan HH. 2004. Pseudo-periodic partitions of biological sequences. *Bioinformatics* **20:** (3) 295.

Luan Y, Li H. 2004. Model-based methods for identifying periodically expressed genes based on time course microarray gene expression data. *Bioinformatics* **20:** (3) 332.

Magnin J, Masselot A, Menzel C, Colinge J. 2004. OLAV-PMF: A novel scoring scheme for high-throughput peptide mass fingerprinting. *J Proteome Res* **3:** (1) 55.

Manduchi E, Grant GR, He H, Liu J, Mailman MD, Pizarro AD, Whetzel PL, Stoeckert CJ. 2004. RAD and the RAD study-annotator: An approach to collection, organization and exchange of all relevant information for high-throughput gene expression studies. *Bioinformatics* **20:** (4) 452.

Murphy K, Raj T, Winters RS, White PS. 2004. me-PCR: A refined ultrafast algorithm for identifying sequence-defined genomic elements. *Bioinformatics* **20:** (4) 588.

Nilsson D, Andersson B. 2004. A graphical tool for parasite genome annotation. *Comput Methods Programs Biomed* **73:** (1) 55.

Ovcharenko I, Loots GG, Hardison RC, Miller W, Stubbs L. 2004. zPicture: Dynamic alignment and visualization tool for analyzing conservation profiles. *Genome Res* **14:** (3) 472.

Serra R, Villani M, Semeria A. 2004. Genetic network models and statistical properties of gene expression data in knock-out experiments. *J Theor Biol* **227:** (1) 149.

Spiro PA, Macura N. 2004. A local alignment metric for accelerating biosequence database search. *J Comput Biol* **11:** (1) 61.

Spitzer M, Fuellen G, Cullen P, Lorkowski S. 2004. VisCoSe: Visualization and comparison of consensus sequences. *Bioinformatics* **20:** (3) 433.

Thollesson M. 2004. LDDist: A Perl module for calculating LogDet pair-wise distances for protein and nucleotide sequences. *Bioinformatics* **20:** (3) 416.

Wu CH, Huang HZ, Nikolskaya A, Hu ZZ, Barker WC. 2004. The iProClass integrated database for protein functional analysis. *Comput Biol Chem* **28:** (1) 87.

Xue W, Wang J, Shen ZR, Zhu HQ. 2004. Enrichment of transcriptional regulatory sites in non-coding genomic region. *Bioinformatics* **20:** (4) 569.

Yabuki Y, Mukai Y, Swindells MB, Suwa M. 2004. GENIUS II: A high-throughput database system for linking ORFs in complete genomes to known protein three-dimensional structures. *Bioinformatics* **20:** (4) 596.

Zhou X, Wang X, Dougherty ER, Russ D, Suh E. 2004. Gene clustering based on clusterwide mutual information. *J Comput Biol* **11:** (1) 147.

Copyright © 2004 John Wiley & Sons, Ltd. 
